# Deleterious Effects of an Air Pollutant (NO_2_) on a Selection of Commensal Skin Bacterial Strains, Potential Contributor to Dysbiosis?

**DOI:** 10.3389/fmicb.2020.591839

**Published:** 2020-12-08

**Authors:** Xavier Janvier, Stéphane Alexandre, Amine M. Boukerb, Djouhar Souak, Olivier Maillot, Magalie Barreau, Frantz Gouriou, Catherine Grillon, Marc G. J. Feuilloley, Anne Groboillot

**Affiliations:** ^1^Laboratory of Microbiology Signals and Microenvironment LMSM EA 4312, University of Rouen-Normandy, Normandy-University, Evreux, France; ^2^Laboratory of Polymers, Biopolymers and Surfaces UMR CNRS 6270, University of Rouen-Normandy, Normandy-University, Mont-Saint-Aignan, France; ^3^Aerothermic and Internal Combustion Engine Technological Research Center, Saint-Etienne-du-Rouvray, France; ^4^Center for Molecular Biophysics, UPR CNRS 4301, Orléans, France

**Keywords:** air pollution, nitrogen dioxide (NO_2_), skin microbiota, *Staphylococcus*, *Pseudomonas*, *Corynebacterium*, AFM (atomic force microscope), 3-nitrotyrosine(3-NT)

## Abstract

The skin constitutes with its microbiota the first line of body defense against exogenous stress including air pollution. Especially in urban or sub-urban areas, it is continuously exposed to many environmental pollutants including gaseous nitrogen dioxide (gNO_2_). Nowadays, it is well established that air pollution has major effects on the human skin, inducing various diseases often associated with microbial dysbiosis. However, very few is known about the impact of pollutants on skin microbiota. In this study, a new approach was adopted, by considering the alteration of the cutaneous microbiota by air pollutants as an indirect action of the harmful molecules on the skin. The effects of gNO_2_ on this bacterial skin microbiota was investigated using a device developed to mimic the real-life contact of the gNO_2_ with bacteria on the surface of the skin. Five strains of human skin commensal bacteria were considered, namely *Staphylococcus aureus* MFP03, *Staphylococcus epidermidis* MFP04, *Staphylococcus capitis* MFP08, *Pseudomonas fluorescens* MFP05, and *Corynebacterium tuberculostearicum* CIP102622. Bacteria were exposed to high concentration of gNO_2_ (10 or 80 ppm) over a short period of 2 h inside the gas exposure device. The physiological, morphological, and molecular responses of the bacteria after the gas exposure were assessed and compared between the different strains and the two gNO_2_ concentrations. A highly significant deleterious effect of gNO_2_ was highlighted, particularly for *S. capitis* MFP08 and *C. tuberculostearicum* CIP102622, while *S. aureus* MFP03 seems to be the less sensitive strain. It appeared that the impact of this nitrosative stress differs according to the bacterial species and the gNO_2_ concentration. Thus the exposition to gNO_2_ as an air pollutant could contribute to dysbiosis, which would affect skin homeostasis. The response of the microbiota to the nitrosative stress could be involved in some pathologies such as atopic dermatitis.

## Introduction

Skin is the largest organ of the human body and acts as an interface with the environment. This complex and natural physico-chemical barrier provides a suitable environment for the development of a wide range of microorganisms such as bacteria, fungi, and viruses. This ecosystem constitutes an essential first line of host defense against injury and infection (Grice and Segre, 2011; Kong and Segre, 2012). Bacteria appear as the most abundant microorganisms on the skin (Oh et al., 2014) and their concentration has been evaluated as 10^6^/cm^2^ (Grice et al., 2008). In healthy individuals, this bacterial population is harmless or beneficial to the host, providing a protection against pathogens and playing a central role in skin homeostasis (Rosenthal et al., 2011; Baldwin et al., 2017). The diversity, composition, and stability of bacterial skin microbiota are influenced and modulated by intrinsic factors, as age or gender (Flores et al., 2014; Si et al., 2015; Oh et al., 2016) but also environmental factors like hygiene practices or ambient air… (Rosenthal et al., 2011; Kim et al., 2018). Thus, unbalanced microbial state or dysbiosis can be induced resulting in some common skin disorders such as atopic dermatitis, rosacea, psoriasis, or acne (Schommer and Gallo, 2013; Baldwin et al., 2017; Byrd et al., 2018). Among the environmental factors, atmospheric pollutants, including nitrogen dioxide (NO_2_), are considered to cause significant skin disturbances. Nowadays, it is well established that atmospheric pollutants can affect the skin through direct mechanisms such as generation of free radicals, induction of inflammatory processes or the activation of the aryl hydrocarbon receptor (AhR) (Drakaki et al., 2014; Mancebo and Wang, 2015; Araviiskaia et al., 2019). A new approach consists in considering the alteration of the cutaneous microbiota by pollutants as an indirect action of these pollutants on the skin. Few studies have investigated the effects of pollutants on skin microbiota (He et al., 2006; Burns et al., 2019).

An increase in NO_2_ atmospheric concentration has been correlated with an increase of lentigo in Caucasian and Asian people (Hüls et al., 2016) and an occurrence of atopic dermatitis in children in Germany (Morgenstern et al., 2008). Anthropogenic emissions of NO_2_ into the atmosphere are mainly due to road transport and energy production. NO_2_ is also known to be toxic by inhalation, increasing cardiovascular, and respiratory diseases (Latza et al., 2009). This makes NO_2_ one of the five atmospheric pollutants of greatest concern to human health. Air quality standards defined by EU ambient air quality directives and World Health Organization (WHO) air quality guidelines establish a limit value of 200 μg/m^3^ NO_2_ (0.11 ppm) as hourly average limit. The European Environment Agency sets an alert threshold of 400 μg/m^3^ (0.21 ppm). However, many urban areas have ambient air quality that regularly exceeds these guidelines (European Environment Agency, 2019). In addition, NO_2_ peaks concentrations were recorded transiently in tunnel at 1 ppm in Australia (Sydney) (Martin et al., 2016) and in extreme conditions up to 6.9 ppm in France (Rouen) (Morin et al., 2009). These values are probably higher in some areas although all data are not public.

At the cellular level, reactions with NO_2_ lead to nitrosative stress with the production of reactive nitrogen species (RNS) responsible for alterations of proteins, lipids, and nucleic acids (Pacher et al., 2007; INERIS, 2011). In contrast, and to our best knowledge, the effect of the gaseous NO_2_ was never evaluated on the skin microbiota.

The aim of this study was to investigate the impact of gaseous NO_2_ (gNO_2_) on various commensal bacterial strains. Five representative bacterial species of the cutaneous microbiota covering the principal phyla were selected. In order to mimic the real-life contact of skin bacteria with air gaseous pollutants, a gas exposure device was developed from a prototype designed to investigate the effect of diesel engine exhaust on *in vitro* cell culture (Asimakopoulou et al., 2011). In daily life, the skin and its microbiota are subjected to low concentrations of NO_2_ but over very long periods of time, with occasional peaks of pollution. It is therefore very tricky to simulate such exposure. In this work, the real situation was approximated by a simple exposure to high concentrations (10 and 80 ppm) of gNO_2_ over a short period of time (2 h). The physiological, morphological, and molecular responses of the bacteria to gNO_2_ were investigated.

## Materials and Methods

### Bacterial Strains and Culture Conditions

**FIGURE 1:**
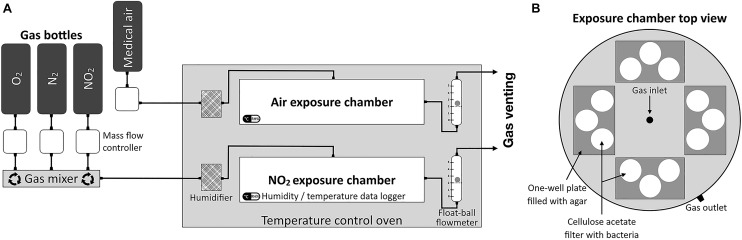
Schematic representation of gas exposure device. **(A)** NO_2_, N_2_, and O_2_ were mixed in a gas mixer to obtain 80 or 10 ppm of gNO_2_ and maintain the 20% O_2_ concentration at a flow rate of 2 L/min in the NO_2_ exposure chamber. The gas flow passed through a humidifier containing 2% copper sulfate solution (CuSO_4_) allowing to maintain the relative humidity (RH%) up to 80%. RH% and temperature were controlled using data logger during the 2 h gas exposure. At each chamber outlet, a ball-float flowmeter allowed to monitor the gas flow. **(B)** Top view of a schematic representation of exposure chamber. Each exposure chamber can contain four agar one-well plates with three cellulose acetate membrane filters each.

Strains used in this study and their origins are listed in Supplementary Table 1. *Staphylococcus aureus* MFP03, *Staphylococcus epidermidis* MFP04, *Staphylococcus capitis* MFP08, and *Pseudomonas fluorescens* MFP05 were isolated from the skin of healthy human volunteers (Hillion et al., 2013). *S. aureus*, *S. epidermidis*, and *S. capitis* were used as representative members of the Firmicutes phylum and *P. fluorescens* as a model of cutaneous proteobacterium. *Corynebacterium tuberculostearicum* CIP102622, originating from Pasteur institute collection, was included in this study as a member of Actinobacteria, one of the three major phyla of skin microbiota (Grice and Segre, 2011). Bacteria were grown overnight at 28°C for *P. fluorescens* and at 37°C for other species with shaking (180 rpm). Luria-Bertani (LB) medium was used as culture media for all bacteria and for most experiments, except for *C. tuberculostearicum* which was cultivated in modified LB medium supplemented with 1% Tween 80 (LBT80 medium) (Becton Dickinson, Heidelberg, Germany). From overnight cultures, fresh medium was inoculated (OD_580_ = 0.08) to obtain cultures at mid-exponential phase (OD_580_ ∼ 0.6). A defined volume of bacterial cultures was spread on each cellulose acetate membrane filter (pore size 0.2 μm, diameter 47 mm, Sartorius Biolab Products, Göttingen, Germany) impregnated with LB or LBT80 and pre-disposed on LB or LBT80 agar. Filters were incubated at optimum growth temperature of each testing bacteria during 4.1 generation time, resulting in a single layer bacterial population. This optimal incubation time was determined by preliminary studies. After incubation, filters with bacteria were placed on agar in one-well dishes (127.8 × 85.5 mm, Thermo Scientific Nunc, Roshester, NY, United States) and transferred into the gas exposure device (Figure 1A).

### Exposure Protocol to Gaseous NO_2_

The gas exposure device (Ghaffari et al., 2005; Asimakopoulou et al., 2011) consisted of two sterile cylindrical Teflon chambers (Figure 1B), one for gNO_2_ exposure and another for air exposure. These exposure chambers were used in parallel in a static incubator and maintained at the optimal growth temperature of the bacterial species. In order to mimic the interaction of gas with bacteria on the skin surface, bacteria on cellulose acetate filter were exposed to gNO_2_ or air under a continuous flow during 2 h. Experiments were realized using gNO_2_ 10 or 80 ppm. NO_2_, N_2_, and O_2_ were provided by different gas bottles (Air Liquide, Mitry-Mory, France) and air by a medical grade air compressor. Gas flow rates were controlled by mass flow regulators (Alicat Scientific, Inc., Tucson, AZ, United States) in order to obtain the desired concentration of gNO_2_, and 20% of O_2_, with a global flow rate of 2 L/min. Temperature and relative humidity data were monitored inside the exposure chambers using a data logger (Lascar Electronics, Inc., Erie, PA, United States). After gas exposure, bacteria were resuspended by gentle agitation for 20 s per filter into physiological water and used for the further analysis.

### Cultivability

Bacterial numeration was performed by the viable count technique after decimal dilution in physiological water and spreading onto LB or LBT80 agar with incubation at the optimal growth temperature. Results are expressed as CFU/Filter of suspension obtained after exposure. The cultivability was assessed by calculating the base 10 logarithm of the ratio of the count after exposure to air against that after exposure to gNO_2_ as shown below:

$$\mathrm{LOG}\,\mathrm{reduction}={\text{log}}_{10}\,\left(\frac{\mathrm{CFU}\,\mathrm{per}\,\mathrm{filter}\,\mathrm{after}\,\mathrm{Air}\,\mathrm{exposure}}{\mathrm{CFU}\,\mathrm{per}\,\mathrm{filter}\,\mathrm{after}\,{\mathrm{NO}}_{2}\,\mathrm{exposure}}\right)$$

### Growth Kinetics

Ninety-six-well Bioscreen microplates (Thermo Fisher Scientific, Roshester, NY, United States) were inoculated with bacterial suspensions in LB or LBT80 at an initial OD_580_ of 0.08. Bacterial suspensions were directly obtained from bacteria grown on cellulose acetate filters exposed to air or gNO_2_ and recovered as previously described. Growth kinetics were measured at OD_580_ under 15 min interval using a Bioscreen C automated microplate reader (Labsystems Oy, Helsinki, Finland) allowing determination of generation time and lag phase length.

### Exo-Enzymatic Activities

Exo-enzymatic activities were tested by culture on specific media. The lipolytic activity (lipase production) was investigated on tributyrin agar plates for *Staphylococci* (Xie, 2012) and rhodamine B agar plates for *P. fluorescens* MFP05 and *C. tuberculostearicum* CIP102622 (Kouker and Jaeger, 1987). Esterase production was assayed on trypcase soy agar (TSA) medium (Sigma-Aldrich, St Quentin Fallavier, France) complemented with 1% Tween 80, 1% CaCl_2_, and 1% phenol red (Plou et al., 1998). Caseinase (protease) activity was tested on TSA with 10% semi-skimmed milk (Riffel and Brandelli, 2006). Determination of the lecithinase (phospholipid hydrolase) activity was performed on TSA with 5% egg yolk agar (McClung and Toabe, 1947).

### Motilities Assays

*Pseudomonas fluorescens* MFP05 was the unique motile species in the series of selected cutaneous bacteria. This bacterial species can move by swimming, swarming, and twitching depending on the surface. These different modes of motility involve flagella, type IV pili, and surfactant secretion (Henrichsen, 1972; Déziel et al., 2001). *P. fluorescens* swimming was tested by surface inoculation of LB medium-agar 0.3% (wt/vol), swarming by using LB medium-agar 0.6% (wt/vol) and twitching by inoculation in depth LB medium-agar 1% (wt/vol) plates, according to Déziel et al. (2001). The diameter of the migration halo was measured after 24 h of incubation at 28°C.

### Biofilm Formation Studies

Biofilm formation was investigated in brain heart infusion (BHI) medium enriched with 2% (wt/vol) glucose for *Staphylococci*, LB medium for *P. fluorescens* MFP05 or LBT80 medium for *C. tuberculostearicum* CIP102622. Bacterial pellets obtained after gas exposure were diluted into 1 ml of fresh medium at OD_580_ = 0.8 and grown in 24-well glass-bottomed microplates (SensoPlate^TM^, Greiner Bio-One, Courtaboeuf, France) for 24 h at their respective optimal growth temperatures. After incubation, biofilms were rinsed twice with sterile physiological water in order to eliminate planktonic bacteria and traces of growth media. Biofilms were stained by adding 5 μM of SYTO 9 green fluorescent nucleic acid stain (Invitrogen, Molecular Probes, Carlsbad, CA, United States) prepared in sterile physiological water and incubated at room temperature for 15 min in the dark and then washed again with physiological water. Biofilm images were acquired with a Zeiss LSM710 confocal laser scanning microscopy (CLSM) (Carl Zeiss, Oberkochen, Germany) using laser excitation at 488 nm, emission band pass 500–550 nm, and an ×63 oil immersion objective. At least five image stacks (image taken every micrometer throughout the biofilm depth) from five independent experiments with two replicates for each experimental condition were used. 3D images were generated using the Zen 2.1 software.^1^ Biofilm average thickness, biovolume, and roughness (Ra) were determined using the COMSTAT2 software^2^ (Heydorn et al., 2000; Vorregaard, 2008).

### Antibiotic Susceptibility Testing

Antimicrobial susceptibility testing was done on Mueller-Hinton II agar (MH-II agar) (Becton Dickinson, Heidelberg, Germany) for all strains except for *C. tuberculostearicum* CIP102622 which was grown on a modified MH-II agar completed with 1% Tween 80. The disk diffusion technique was used according to the EUCAST (Société Française de Microbiologie, 2019). All tested antibiotic disks (Oxoid, Basingstoke, Hampshire, United Kingdom) are listed in Supplementary Table 2. Antibiotics susceptibility profiles were interpreted on the basis of the EUCAST guide (Société Française de Microbiologie, 2019).

### Nitrotyrosine Quantification

3-nitrotyrosine, a nitrosative stress biomarker, was quantified with the nitrotyrosine competitive ELISA kit (Abcam, Cambridge, United Kingdom), usually used for eukaryotic cells. The protocol was adapted for bacteria. After air or gNO_2_ treatments, bacteria cells resuspended from the filter were centrifuged at 12,000 × *g* for 10 min and rinsed thrice in a PBS solution. At the third rinsing step, pellets were recovered in 1 ml of 1× sample extraction and transferred into 1 ml vial with 0.1 mm bead glass (Bertin Instruments, Montigny-le-Bretonneux, France). Bacterial lysis was performed at 4°C using a Precellys24 Bead Beater (6,800 rpm, 3 × 30 s, 120 s break) equipped with a Cryolys cooling system (Bertin Instruments, Montigny-le-Bretonneux, France). Samples were centrifugated and the supernatant was used to determine the 3-nitrotyrosine (3-NT) level according to the manufacturer’s instructions.

### Evaluation of Bacterial Membrane Integrity

Bacterial membrane integrity was investigated by flow cytometry (CytoFlex S flow cytometer, as previously described) after staining of the bacteria with a combination of fluorescent dyes [SYTO 9 and propidium iodide (PI)] (LIVE/DEAD^TM^ BacLight^TM^ Bacterial Viability kit, Invitrogen, Molecular Probes, Carlsbad, CA, United States). Both dyes bind specifically to nucleic acids. Bacterial membranes are permeable to SYTO 9 (green fluorescence) that stains all bacteria, whereas PI (Red fluorescence) only penetrates bacteria with damaged membranes and quenches the SYTO 9 signal. Three populations can be distinguished corresponding to intact green bacteria, permeabilized red and orange bacteria, or unstained cells debris (Léonard et al., 2016). This procedure was impossible to apply with *C. tuberculostearicum* CIP102622 which, as all *Corynebacteria*, is known to be difficult to stain with both dyes, probably because of the specific composition of its cell wall (Neumeyer et al., 2013). Then, *C. tuberculostearicum* CIP102622 was only stained with PI. For each analysis, a positive control was realized using an aliquot of bacterial suspension treated with 100% ethanol for 1 h. SYTO 9 and PI were excited with a 22 mW blue laser (488 nm) and their fluorescence emission were respectively detected at 525/40 nm (green channel) and 690/50 nm (red channel). A total of 200,000 events were recorded at a flow rate of 10 μL.min^–1^. Data were analyzed using the CytExpert Software.

### Flow Cytometry Analysis

Direct analysis by flow cytometry of bacteria without staining provides information regarding the bacterial morphology and structure. The forward scattering (FSC) fraction of light and the fraction of light scattered laterally, known as side scatter (SSC) provide respectively an estimation of the cell size and an analysis of the surface heterogeneity, called granularity (Léonard et al., 2016). Measures were performed on 200,000 events per sample recorded with the CytoFlex S flow cytometer (Beckman coulter Life science, Indianapolis, IN, United States) and the data were analyzed with the internal CytExpert Software.

### Atomic Force Microscopy

Atomic Force Microscopy (AFM) imaging was performed by using a Nanoscope III Multimode microscope (Veeco instrument, Santa Barbara, CA, United States) for *S. capitis* MFP08 or a Nanoscope 8 Multimode microscope (Bruker Nano Surfaces, Santa Barbara, CA, United States) for *S. aureus* MFP03, *S. epidermidis* MFP04, *P. fluorescens* MFP05, and *C. tuberculostearicum* CIP102622. In all cases, a 100 μm piezoelectric scanner was employed. Imaging was achieved in air using the contact mode for all bacteria except *S. aureus* MFP03 for which the PeakForce^®^ mode was used. In the contact mode, the cantilevers, characterized by a low spring constant of 0.06 N/m, were equipped with a classical pyramidal NiSi tip. In this condition, all measurements were performed with the feedback loop on (constant force from 10^–9^ to 10^–8^ N). In the PeakForce^®^ mode, a tapping cantilever with a spring constant of 40 N/m and a classical Si tip was used. Images were obtained with a peak force tapping frequency of 2 kHz with the auto-amplitude on. All images are presented in the height mode and are top view. Flatten and three points leveling operations were realized using the Gwyddion AFM software.^3^ Images were also processed using a local contrast filter (kernel size 2 px, blending depth 2, and weight 1) in order to visualize the bacteria surface. Then, the local contrast image was overlayed to the topographic image using the GIMP software^4^ in order to get more meaningful image.

### Statistical Analysis

All experiments were carried out at least three times. For analysis and graphical presentation, GraphPad Prism^®^ Software (V8.3.1, San Diego, CA, United States) was used. Shapiro–Wilk normality test was used to verify normality of the data and a ratio paired *t*-test or a paired *t*-test was applied to compare air and gNO_2_ conditions. To compare the results obtained with the different strains, one-way ANOVA and Tukey’s multiple comparison were performed. To evaluate the correlation between different parameters, a Pearson correlation test was used. A significant difference was considered as (^∗^) for *P* < 0.05, (^∗∗^) for *P* < 0.01, and (^∗∗∗^) for *P* < 0.001.

## Results

### gNO_2_ Reduces the Cultivability of Cutaneous Bacteria

**FIGURE 2:**
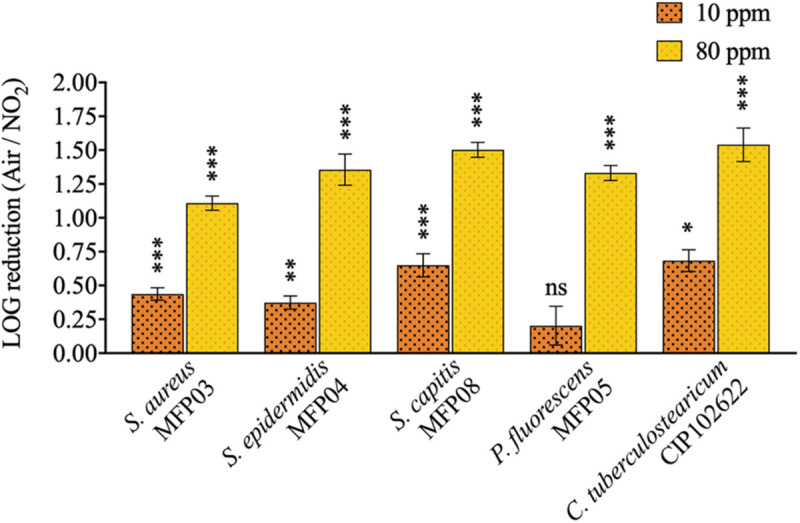
Cultivability loss induced by 10 or 80 ppm gNO_2_. Results are displayed as LOG reduction, i.e., the base 10 logarithm of the ratio of cultivability (CFU/filter) induced by air and that induced by gNO_2_ and that induced by air. Error bars show standard error of the mean of independent treatments (*n* ≥ 3). Statistical significance was calculated by a ratio paired *t*-test. (^∗^) for *P* < 0.05, (^∗∗^) for *P* < 0.01, and (^∗∗∗^) for *P* < 0.001.

The decrease of bacterial cultivability after exposure to gNO_2_ 10 and 80 ppm in comparison to air is presented in Figure 2 and Supplementary Figure 1. A 2 h exposure to gNO_2_ 80 ppm resulted in a mean cultivability loss of 1.36 LOG units for the five strains, with a maximum loss of 1.54 and 1.50 LOG units for *C. tuberculostearicum* and *S. capitis*, respectively, and a minimum of 1.11 LOG units for *S. aureus*. The cultivability decrease varied significantly between strains at 10 ppm (*P* = 0.0055) and 80 ppm (*P* = 0.0003). A lower LOG reduction (<1 LOG) was observed for 10 ppm, with an average cultivability loss of 0.47 LOG units and a maximum loss of 0.68 and 0.65 LOG units for *C. tuberculostearicum* and *S. capitis*, respectively. Surprisingly, after a 10 ppm exposure, *P. fluorescens* showed no significant loss of cultivability, unlike the other four bacterial species.

### gNO_2_ Affects the Growth Kinetics of Cutaneous Bacteria

**FIGURE 3:**
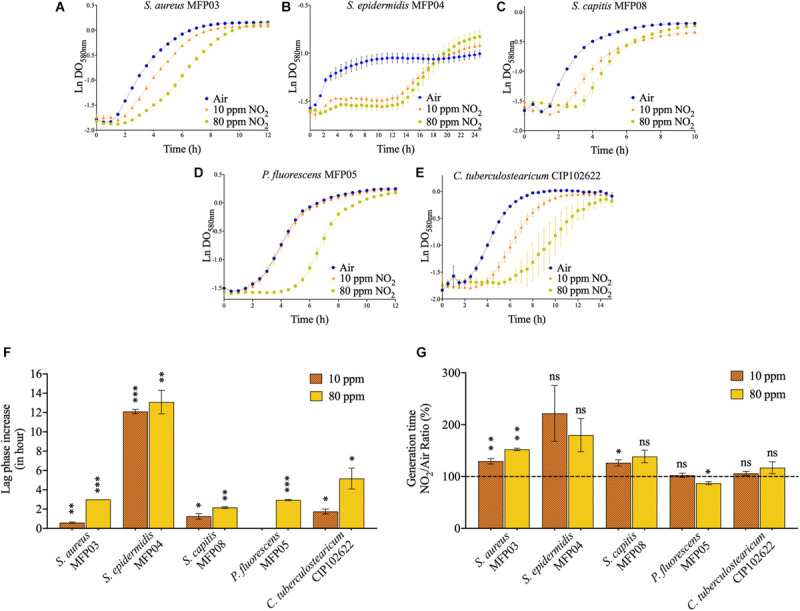
Bacterial growth kinetics altered by gNO_2_. Growth curves of **(A)** *S. aureus* MFP03, **(B)** *S. epidermidis* MFP04, **(C)** *S. capitis* MFP08, **(D)** *P. fluorescens* MFP05, and **(E)** *C. tuberculostearicum* CIP102622 after air or gNO_2_ (10 and 80 ppm) exposure. **(F)** Lag phase increase and **(G)** NO_2_/Air ratio of generation time after gNO_2_ exposure. Error bars show standard error of the mean of independent treatments (*n* ≥ 3). Statistical significance was calculated by a paired *t*-test for **(F)** and a ratio paired *t*-test for **(G)**. (^∗^) for *P* < 0.05, (^∗∗^) for *P* < 0.01, and (^∗∗∗^) for *P* < 0.001.

Exposure to gNO_2_ altered the growth kinetics of *S. aureus*, *S. epidermidis*, *S. capitis*, *P. fluorescens*, and *C. tuberculostearicum* (Figures 3A–E, respectively). A 80 ppm exposure increased the lag phase of the five strains, from a minimum of +2.16 h for *S. capitis* to a maximum of +13 h for *S. epidermidis*, showing a strain-related effect of gNO_2_ (*P* = 0.0165). At 10 ppm, the lag phase increase was less pronounced and even absent in *P. fluorescens*, but always variable between the strains (*P* = 0.0098) (Figure 3F). Except for *P. fluorescens*, the generation time generally tended to increase or remain unchanged, depending on the strain, for both gNO_2_ tested concentrations (*P* < 0.0001) (Figure 3G). *S. epidermidis*, unlike the other S*taphylococci*, is difficult to grow in LB culture medium. This may explain the high variability of the obtained results as well as the significant increase in generation time and latency phase observed after exposure to gNO_2_, compared to other species.

### Absence of Effect on the Bacterial Motility and Exo-Enzymes Secretion

*Pseudomonas fluorescens* was the unique motile microorganism selected in the present study. This bacterium is known to be able to use different types of motility according to the culture conditions, namely swimming, swarming, and twitching. Bacteria exposed to gNO_2_ 80 ppm remained capable to migrate using the three types of movement. The diffusion distance and aspect of halo were unchanged (Supplementary Table 3). However, the migration was delayed by 24 h compared to bacteria exposed air.

The lipase, phospholipid hydrolase (lecithinase), protease (caseinase), and esterase exo-enzymatic activities of the five selected cutaneous bacterial strains were tested in the absence or presence of gNO_2_ 80 ppm. These activities were unchanged between control and gNO_2_ exposed microorganisms although a 24 to 48 h delay was observed for appearance of the phenotype in comparison to bacteria in contact with air (Supplementary Table 4).

### gNO_2_ Affects Differently the Biofilm Formation of Cutaneous Bacteria

**FIGURE 4:**
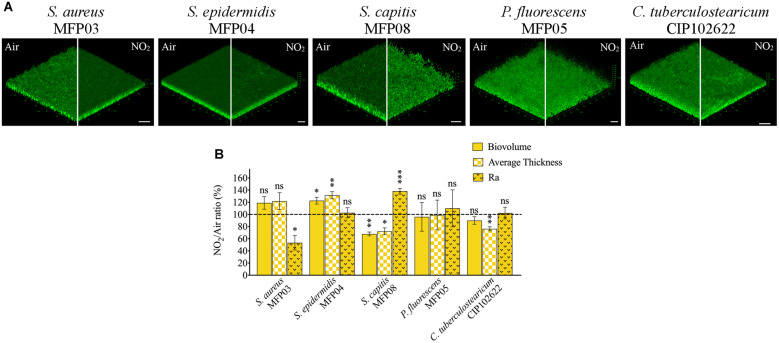
Effect of 80 ppm gNO_2_ on biofilm formation. **(A)** CLSM images of 24 h biofilms formed by bacteria after air or 80 ppm gNO_2_ exposure. For each biofilm, a 3D view along the *x*, *y*, and *z-*axes is displayed. Images show representative data from at least five independent biofilm assays. Scale bars = 20 μm. **(B)** COMSTAT2 image analyses were performed to determine the ratio (NO_2_/Air) of biovolume (μm^3^/μm^2^), average thickness (μm), and roughness coefficient (Ra). Error bars show standard error of the mean of independent treatments (*n* ≥ 5). Statistical significance was calculated by a ratio paired *t*-test. (^∗^) for *P* < 0.05, (^∗∗^) for *P* < 0.01, and (^∗∗∗^) for *P* < 0.001.

The effect of gNO_2_ 80 ppm on biofilm formation was studied by CLSM in order to visualize the different morphological alterations (Figure 4A). Image analysis was leading to calculate the mean biovolume, roughness, and average thickness of the biofilm (Figure 4B). gNO_2_ had limited effects on *S. aureus* biofilms formation and only the mean roughness was significantly decreased. Conversely, in the case of *S. epidermidis* the roughness was not modified by gNO_2_ but the biovolume and average thickness of the biofilm were increased. *S. capitis* showed another reaction to gNO_2_ with a decrease of biofilm biovolume and thickness and an important rise of the roughness. For *P. fluorescens* no significant effect of gNO_2_ on the biofilm formation activity was observed. *C. tuberculostearicum* was also marginally affected by gNO_2_ with just a decrease of the average thickness.

### Absence of Effect on Antibiotic Susceptibility

The sensitivity of our five bacterial strains to a wide range of antibiotics covering the principal active families was tested as recommended by the EUCAST. No effect of gNO_2_ 80 ppm pretreatment was observed on the antibiotic susceptibility. All antibiotics susceptibility profiles are available in Supplementary Table 2.

### gNO_2_ Increases the Level of 3-Nitrotyrosine in All Treated Bacteria

**FIGURE 5:**
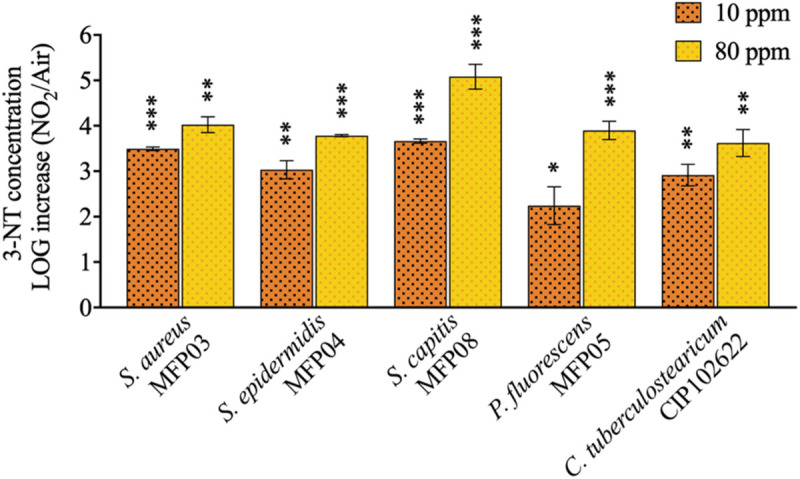
Quantification of 3-NT-modified proteins level induced by 10 or 80 ppm gNO_2_. Results are displayed as the LOG increase, i.e., the base 10 logarithm of the ratio between the 3-NT concentration induced by gNO_2_ and that induced by air. Error bars show standard error of the mean of independent treatments (*n* ≥ 3). Statistical significance was calculated by a ratio paired *t*-test. (^∗^) for *P* < 0.05, (^∗∗^) for *P* < 0.01, and (^∗∗∗^) for *P* < 0.001.

The level of 3-NT is usually employed as marker of protein damage induced by RNS in eukaryotic cells and results from the action of the peroxynitrite ion (ONOO^–^) generally generated from nitric oxide (NO) (Pacher et al., 2007; Teixeira et al., 2016). This method was successfully applied to the global evaluation of the effect of gNO_2_ on bacterial proteins. The level of 3-NT increased significantly for all the bacterial species tested after exposure to gNO_2_ 10 or 80 ppm (Figure 5). For the two concentrations, the effect of gNO_2_ was species-depended (*P* = 0.0208 for 10 ppm and 0.0028 for 80 ppm). At gNO_2_ 80 ppm, *S. capitis* showed a significantly greater increase (5.08 LOG units) compared with *S. aureus* (*P* = 0.0232), *P. fluorescens* (*P* = 0.0109), *S. epidermidis* (*P* = 0.0097), and *C. tuberculostearicum* (*P* = 0.0039). At 10 ppm, the least affected strain was *P. fluorescens* (2.24 LOG units).

### gNO_2_ Increases the Bacterial Membrane Permeability

**FIGURE 6:**
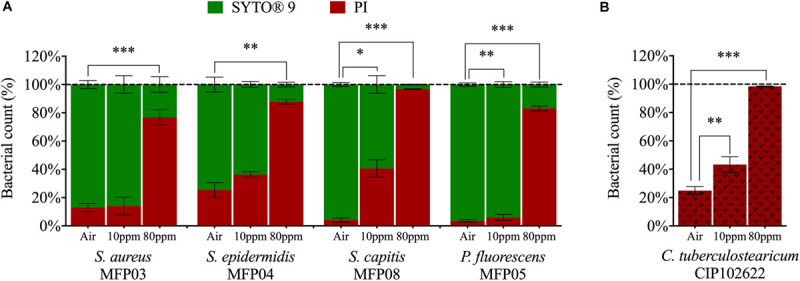
Membrane permeability increase at 10 or 80 ppm gNO_2_. Percentage of PI-permeable bacteria exposed to gNO_2_ compared to that exposed to air using **(A)** the SYTO 9 / PI combination for *S. aureus*, *S. epidermidis*, *S. capitis*, and *P. fluorescens* or **(B)** PI only for *C. tuberculostearicum* due to its unconventional cell wall structure which compromises the labeling with SYTO 9 dye. Error bars show standard error of the mean of independent treatments (*n* ≥ 3). Statistical significance was calculated by a paired *t*-test. (^∗^) for *P* < 0.05, (^∗∗^) for *P* < 0.01, and (^∗∗∗^) for *P* < 0.001.

Membrane permeability was assessed by flow cytometry and labeling with SYTO 9 / propidium iodide (PI) or PI alone. The membrane permeability of all strains was strongly increased after exposure to gNO_2_ 10 or 80 ppm (Figure 6). The percentage of bacteria permeable to PI following exposure to air varied between species (*P* < 0.0001 for 10 ppm and *P* = 0.0331 for 80 ppm). The increase in permeability caused by gNO_2_ was therefore evaluated by considering the difference of percentages measured in gNO_2_ and air exposed bacteria (Table 1). This increase ranged from +61% for *S. aureus* to +90.1% for *S. capitis*. In coherence with the loss of cultivability observed at gNO_2_ 80 ppm, a lower increase of permeability was observed for all bacteria exposed to this concentration of gNO_2_, with a marginal effect on permeability for *S. aureus* (+1.8%) and *P. fluorescens* (+0.99%) (Table 1).

**TABLE 1:** Increase of PI-permeable bacteria percentage after gNO_2_ exposure.

	**PI-permeable bacteria increase (%)^*a*^**				
	**MFP03**	**MFP04**	**MFP08**	**MFP05**	**CIP102622**
10 ppm	7.1 ± 4.1	1.8 ± 4.4	37.1 ± 7.0*	0.99 ± 0.4	24.2 ± 0.8**
80 ppm	61.0 ± 8.7***	71.6 ± 4.6**	90.1 ± 2.8***	80.6 ± 1.8***	72.2 ± 2.6***

^*a*^Data represent the difference of percentage of PI-permeable bacteria between air and gNO_2_ from at least three independent experiments ± standard error of the mean (SEM). Statistical significance was calculated by a paired *t*-test. (*) for *P* <0.05, (**) for *P* < 0.01, and (***) for *P* < 0.001.

### Inter-Species Analyses of the Data

**FIGURE 7:**
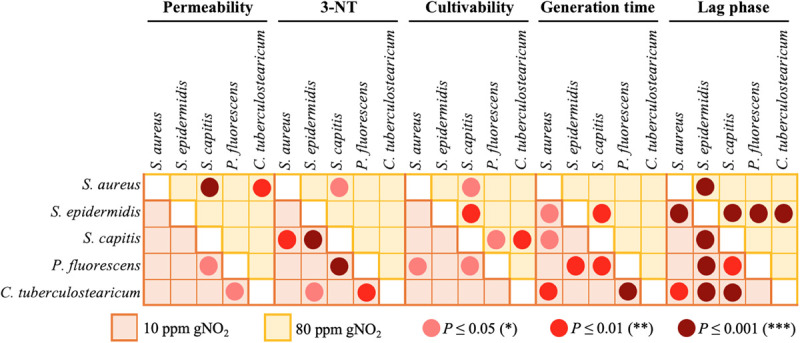
Inter-strain differences. Matrices showing the significance (*P*-values obtained by multiple comparison of strains using the Tukey’s test following one-way ANOVA) of the inter-strain differences induced by 80 (yellow) or 10 ppm (orange) gNO_2_ for the permeability, 3NT-level, culitvability, generation time and lag phase duration. The pink, red, and dark red dots indicate the level of *P*-values significance <0.005, <0.001, and <0.0001, respectively.

As mentioned above, for all the parameters tested, different effect of gNO_2_ were observed according to bacterial species considered and the pollutant concentration. Figure 7 summarizes the *P*-values obtained by the multiple comparison of the strains using the Tukey’s test for all the parameters (except biofilm formation not tested at 10 ppm) and both concentrations. More significant differences appeared at 10 ppm rather that at 80 ppm, probably because 80 ppm induced a saturation effect, masking inter-species variations.

### Envelope Alteration of the Cutaneous Bacteria by gNO_2_

**FIGURE 8:**
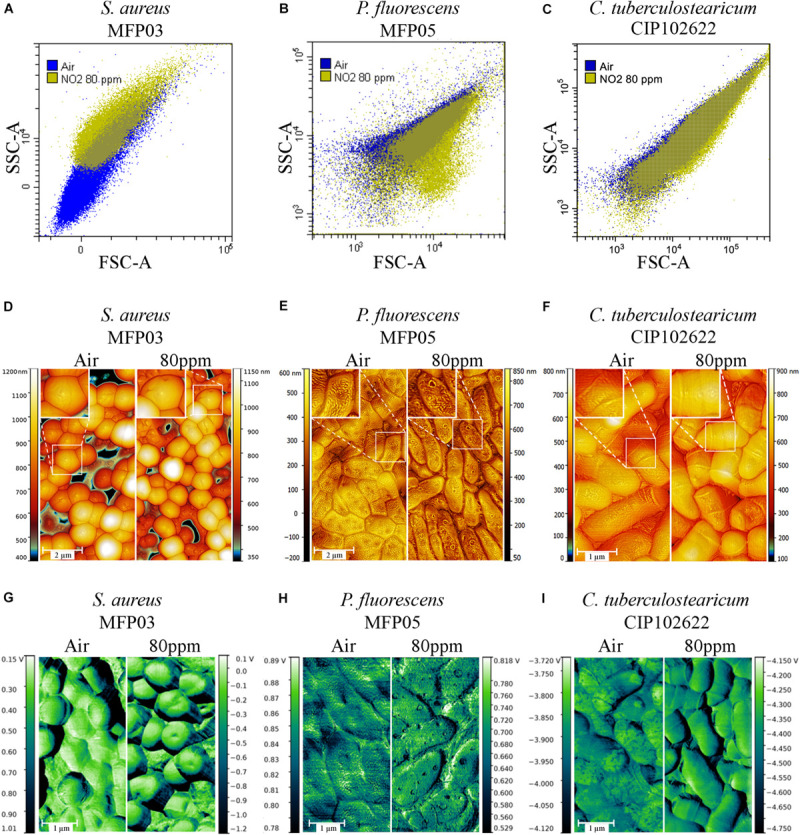
Impact of 80 ppm gNO_2_ on bacterial structure observed by flow cytometry and AFM. Dot plot overlays of FSC and SSC parameters obtained by flow cytometry analysis for air (blue) exposure and 80 ppm gNO_2_ (yellow) exposure of **(A)** *S. aureus* MFP03, **(B)** *P. fluorescens* MFP05, and **(C)** *C. tuberculostearicum* CIP102622. Topographic images with overlaid local contrast images of **(D)** MFP03, **(E)** MFP05, and **(F)** CIP102622. Square in the upper left corner of each image corresponds to a zoom on the bacterial surface. **(G–I)** Representation of the frictional forces of MFP03, MFP05, and CIP102622, respectively.

Direct analysis by flow cytometer without staining of bacteria after gas exposure was performed for the two gNO_2_ concentrations: 10 and 80 ppm. Only the 80 ppm concentration induced a modification of the FSC/SSC dot plot clouds (Figures 8A–C). Changes in size and granularity appear differently depending on the species and three different behaviors were observed. In the case of *S. aureus* (Figure 8A) and the other studied *Staphylococci* (Supplementary Figures 2A,B), an increase in the mean cell size and complexity was observed. Concerning *P. fluorescens*, exposure to gNO_2_ 80 ppm induced an increase in mean cell size and a decrease in complexity (Figure 8B). *C. tuberculostearicum* showed another behavior, characterized by a minor size increase and an absence of cell complexity variation (Figure 8C).

In order to complete the flow cytometry analysis and visualize possible alterations of the bacterial envelope, an AFM analysis was performed. In topographic images, depression areas were observed on the surface of many *S. aureus* cells exposed to 80 ppm gNO_2_ (Figure 8D). Similar alterations were found in higher occurrence in *S. epidermidis* and *S. capitis* (Supplementary Figures 3A,B). Another type of alteration, characterized by appearance of pore-like structures, was observed on the surface of *P. fluorescens* exposed to gNO_2_ 80 ppm. In addition, whereas control bacteria showed wavelet structures, this pattern was totally absent after exposure to gNO_2_ 80 ppm (Figure 8E). Interestingly, no visible surface alteration was observed in gNO_2_ treated *C. tuberculostearicum* (Figure 8F). Measurement of frictional forces revealed no difference in *S. aureus* (Figure 8G) and *P. fluorescens* (Figure 8H) after gNO_2_ exposure. In contrast, chemically different surface domains observed in control *C. tuberculostearicum* disappeared in bacteria exposed to gNO_2_ (Figure 8I), suggesting that gNO_2_ induced a rearrangement of the cell wall.

## Discussion

Reactive nitrogen species, i.e., molecules generated mainly from NO, are often considered as atmospheric pollutants such as NO_2_, its main representative in ambient air. However, NO is synthetized *in vivo* by cells and involved at various biological functions such as innate immunity (Bogdan, 2015) or cell signaling (Martínez-Ruiz et al., 2011) including in bacteria (Williams and Boon, 2019). NO and RNS exert in macrophages antimicrobial effects allowing host protection against pathogens (Bogdan, 2015). Some bacteria are known to resist to NO, partly through detoxification enzymes. Consequently this ability occurring particularly in pathogens is considered by itself as a virulence trait (Richardson et al., 2006). The impact of NO on bacteria is widely described in the literature (Bowman et al., 2011). However, studies on this impact have been generally achieved in liquid phase using chemical donors of NO and more rarely by passing NO gas through in the aqueous mixture. This is essential to keep in mind as the effects on bacteria can be markedly different according the way NO is delivered (Bowman et al., 2011). The effect of NO_2_ has been much less investigated in bacteria. As there is no chemical donor for NO_2_, bacteria were mainly plated on agar or membrane surface and directly placed in a gas-swept chamber allowing cell growth in contact with gNO_2_ (Mancinelli and McKay, 1983; Yu et al., 1999; Ghaffari et al., 2005). Surprisingly enough, the impact of this major air pollutant on cutaneous bacteria was not investigated until now. As exposing the skin of human volunteers to a toxic compound such as gNO_2_ which is drastically limited by safety and ethic regulations, in a first step it was preferred to evaluate first the effect of gNO_2_ on bacterial model species of the skin microbiota. Hence, in the present study we decided to investigate the effect of gNO_2_ using five representative bacterial species of the cutaneous microbiota. In order to mimic the effect of this air pollutant on the microbiota of the skin surface, the contact of gNO_2_ with bacterial microcolonies previously grown on a membrane carrier appeared particularly well adapted. Two high gNO_2_ concentrations (10 and 80 ppm) were employed to compensate for the short possible exposure time (2 h) required to avoid artifacts due to dehydration.

In most cases, exposure of *S. aureus*, *S. epidermidis*, *S. capitis*, *P. fluorescens*, and *C. tuberculostearicum* to gNO_2_ led to a significant decrease of cultivability. Similar effects were observed for gNO or gNO_2_ (Mancinelli and McKay, 1983; Yu et al., 1999), where gNO_2_ exhibited highest toxicity. Deleterious effect observed after exposure to other toxic compounds such as metals and antibacterial molecules often results in cultivability loss (Izutani et al., 2011; Hobman and Crossman, 2015). In coherence, the impact of gNO_2_ on skin bacteria was also characterized by an increase of the lag growth phase. The generation time was marginally modified, although *Staphylococci* showed a general increase and *P. fluorescens* a significant decrease at 80 ppm. In contrast, identical maximal optical density at 580 nm (OD_580_) values were reached at the stationary phase. These observations indicate that, at the tested gNO_2_ concentrations, bacteria were submitted to a non-lethal metabolic stress requiring the induction of specific detoxification pathways and adaptation mechanisms (Bertrand, 2019). Regarding RNS, detoxification processes are particularly numerous and consist partly of enzymes able to consume NO, resulting in protection of NO-sensitive proteins (Poole, 2004). Bacterial physiology was deeply altered following gNO_2_ exposure as observed with growth metrics. The question then arose as to whether the physiological functions necessary for colonization and maintenance of the skin commensal bacteria were also affected. Indeed, the exo-enzymes produced by commensal bacteria are necessary for microbial growth on host skin substrates (Koziel and Potempa, 2013; Kwaszewska et al., 2014). The enzymatic activities of the five studied cutaneous bacteria, including lipase, phospholipid hydrolase (lecithinase), protease (caseinase), and esterase remained unchanged after exposure to gNO_2_. However, the delay of response observed for gNO_2_ exposed bacteria should be correlated to the increase of the lag phase and can correspond to the time required for repairing or induction processes or both. Regarding skin bacterial settlement, motility allows cells to interact dynamically with the cutaneous surface and participates to colonization. In *P. fluorescens*, no difference of swimming, swarming or twitching motility was observed with gNO_2_ 80 ppm, although a response delay was also noted, as previously for enzymatic activities. A different result was reported in a previous study in which 45 ppm gNO_2_ were sufficient to reduce the motility of airborne and clinical strains of *P. fluorescens* (Kondakova et al., 2016). However, it is essential to remember that *P. fluorescens* is a species-complex particularly wide and heterogeneous phylogenetically and physiologically and this diversity appears closely related to ecological niches (Bodilis et al., 2004; Scales et al., 2014). Thus, important differences may exist between airborne or clinical isolates compared to our cutaneous commensal low virulence strain such as *P. fluorescens* MFP05 (Hillion et al., 2013). Microcolonies or biofilms also important for skin bacterial colonization, are organized aggregates of microorganisms and constitute a growth pattern associated with surfaces, predominant in the natural environment (Costerton et al., 1999). Although biofilms on skin are often considered to be related to the development of pathogens (Brandwein et al., 2016), commensal bacteria also grow on the skin surface as microcolonies (Somerville and Noble, 1973; Noble, 1975). The biofilm extracellular matrix provides a protection against environmental aggressions (Costerton et al., 1999) that induce stress promoting the conversion of bacteria from planktonic to biofilm mode of development (Jefferson, 2004). *S. aureus* MFP03 did not react to gNO_2_ by an increase of biofilm formation. However, the effect of gNO_2_ on biofilm formation was clearly species dependent, as already observed with NO at low concentration (de la Fuente-Núñez et al., 2013; Arora et al., 2015; Barraud et al., 2015). At the opposite of *S. aureus*, *S. epidermidis* reacted to gNO_2_ by a moderate increase of biofilm formation whereas a significant reduction of the biofilm biovolume and thickness was noted for *S. capitis*. In this strain, the biofilm roughness was markedly increased suggesting a disorganization of the matrix. The effects of gNO_2_ on cutaneous *P. fluorescens* and *C. tuberculostearicum* biofilm formation were limited in contrast to those observed in airborne *P. fluorescens* which exhibited a high increase in biofilm thickness (Kondakova et al., 2016). Globally, the impact of gNO_2_ on exo-enzymes production, motility, and biofilm formation suggests that gNO_2_ should have limited influence on the initial steps of cutaneous bacteria implantation on skin.

Since gNO_2_ induced physiological changes visualized by cultivability loss and longer lag phase, it was necessary to examine the mode of action of gNO_2_ at the molecular level. The absence of impact of gNO_2_ on antibiotics sensitivity of studied cutaneous bacteria suggests that gNO_2_ and antibiotics act through different mechanisms. Conversely, under similar conditions of exposure, in clinical and airborne strains of *P. fluorescens*, gNO_2_ induces an overexpression of genes encoding for the RND efflux pumps MexEF-OprN causing an increase in resistance to ciprofloxacin and chloramphenicol (Kondakova et al., 2016). In fact, as observed for enzymatic activities, motility, and antibiotic susceptibility, cutaneous bacteria appear to share a common high metabolic resilience to gNO_2_. In order to further investigate the mechanism of gNO_2_ action on bacteria, the cytoplasmic level of 3-nitrotyrosine (3-NT) was measured. This compound results usually from the interaction of tyrosine residue with RNS, particularly peroxynitrite (ONOO^–^), and is a biomarker of protein alteration following nitrosative stress (Pacher et al., 2007; Teixeira et al., 2016). A significant increase in 3-NT level was observed in all studied bacteria, even after exposure to 10 ppm gNO_2_. It is interesting to note that this dose of gNO_2_, which affected the growth kinetic and cultivability of most bacterial species, was clearly sub-maximal since the ratio of 3-NT was significantly higher in bacteria exposed to 80 ppm gNO_2_. Nevertheless, these results indicate that even at the 10 ppm, gNO_2_ induced significant alterations of bacterial proteins. Thus, from all the tested parameters to evaluate the effect of gNO_2_, 3-NT level appears as the most sensitive. In addition, flow cytometry revealed an augmentation of membrane permeability under the action of gNO_2_ in all bacteria. This increase ranged from +61% in *S. aureus* to +90.1% in *S. capitis* at 80 ppm of NO_2_ and was probably sufficient to decrease the transmembrane pressure and affect ion exchanges and transmembrane electric potential. Moreover, a statistical analysis highlighted a significant correlation (*P* < 0.0095; *R*^2^ = 0.5679; Supplementary Figure 4A) between the increase of membrane permeability and 3-NT level. In fact, the raise of tyrosine residues nitration, may be associated to an increase of gNO_2_ influx into the cells. The high percentage of PI-permeable cells was also correlated to a loss of cultivability after gNO_2_ exposure (*P* < 0.0001; *R*^2^ = 0.7252; Supplementary Figure 4B). This is logical since permeability is an indicator of viability. The increase of 3-NT level was furthermore linked to the loss of cultivability (*P* < 0.0118; *R*^2^ = 0.5899; Supplementary Figure 4C). Consequently, this loss of cultivability may be attributed to an increase of membrane permeability, as well as the deleterious effects of gNO_2_ on proteins and other molecules. In view of this increase in membrane permeability, a morphological analysis of bacteria was relevant.

As demonstrated by flow cytometry and AFM, all cutaneous bacterial species used in this study reacted to gNO_2_ by morphological modifications of their envelope. *S. aureus*, *S. capitis*, and *S. epidermidis* showed similar reactions whereas bacteria of other *genera*, i.e., *C. tuberculostearicum* and *P. fluorescens*, were differently affected. These discrepancies should be related to the structure of the bacterial envelope. *Staphylococci* have a rigid cell wall, essentially formed of peptidoglycan covalently linked to teichoic acid, which can account for 50% of the total mass and lipoteichoid acid connecting to phospholipids of the plasma membrane (Malanovic and Lohner, 2016; Rohde, 2019). After exposure to gNO_2_, all *Staphylococci* reacted the same way by an increase of the mean cell size, surface complexity and the presence of depression areas. In these bacteria, the cell wall is essential to withstand the high internal turgor pressure (25 atm) and any defect leads to a decrease of viability (Giesbrecht et al., 1998). However, probably because of this high-pressure gradient, direct damages of the cell wall are generally associated to blebs and protrusions, as observed after exposure to penicillin (Giesbrecht et al., 1998). Conversely, the morphology of *Staphylococci* after exposure of gNO_2_, was more coherent with alterations of cell wall organization associated to a decrease of internal pressure. Similar envelope damage was observed when *S. aureus* was in contact with ε-poly-lysine, inducing depressions on the surface of cells due to a destruction of the peptidoglycan structure (Tan et al., 2019). *C. tuberculostearicum*, another Gram-positive bacterium, exhibited no morphological change or surface alteration. However, *Corynebacteria* have a peculiar complex cell wall comprising, in addition to the peptidoglycan envelope, a mycolic acid layer covered with a thick top layer consisting essentially of carbohydrates (Burkovski, 2013; Rohde, 2019). This envelope is then probably resistant enough to withstand the attack of sub-lethal concentrations of gNO_2_. Nevertheless, as revealed by measurement of frictional forces, gNO_2_ altered the organization of *C. tuberculostearicum* surface, presumably through chemical modification of the top external layer. As a Gram-negative bacterium with a thin layer of peptidoglycan covered by a lipid membrane, the reaction of *P. fluorescens* to gNO_2_ was totally different with an increase in mean cell size, a decrease of surface complexity and the appearance of pore-like structures. NO was already shown to modify membrane roughness and leading to pores formation on the surface of *Pseudomonas aeruginosa* (Deupree and Schoenfisch, 2009). Thus, different damages were observed according to cell wall structure associated with permeability increase in both Gram-positive and Gram-negative bacteria. This is showing that gNO_2_ affects the peptidoglycan and lipid bilayer membranes as already described with another antimicrobial compound, namely ε-poly-lysine (Tan et al., 2019). Membrane micro-domains or lipid rafts leads to membrane heterogeneity and could participate in the localized disorganization of the lipid bilayer caused by gNO_2_ (Bramkamp and Lopez, 2015; Lopez and Koch, 2017). This question should require further investigations.

Finally, although all studied cutaneous bacteria were impacted by gNO_2_, major interspecies differences were observed. *S. aureus* was remarkably resistant to gNO_2_ probably through the protection conferred by its cell wall but also by the existence in this species of many NO detoxification systems (Richardson et al., 2006; Grosser et al., 2016). *P. fluorescens* was also very resistant to gNO_2_ 10 ppm but the mechanism may be different as bacteria of the genus *Pseudomonas* are Gram-negative and have a large genome which gives them a high metabolic adaptation potential (Scales et al., 2014). At the opposite, *C. tuberculostearicum* and *S. capitis* appear particularly sensitive to gNO_2_. Extrapolation to the whole cutaneous microbiota should be merged considering the complex interactions between microorganisms. However, the significant differential response observed between bacterial species suggests that gNO_2_ could contribute to a dysbiotic state. It is interesting to note that the trends observed in these different strains corroborate the effects of gNO_2_ on the skin concerning atopic dermatitis, which is clearly increased by NO_2_ pollution (Morgenstern et al., 2008). Severe atopic dermatitis flares are associated to *S. aureus* skin colonization and a concomitant decrease of *S. epidermidis* (Hon et al., 2016; Byrd et al., 2018). Our results show that the development of *S. aureus* is favored by gNO_2_ over other species, particularly in regard of *S. epidermidis*. In the skin, this imbalance could be amplified by the antagonistic action of *S. epidermidis* (Pothmann et al., 2019). In this regard, the cutaneous microbiota appears as a new target for pollutants, and its response could contribute to the deleterious effects of gNO_2_ pollution on the skin.

## Data Availability Statement

The raw data supporting the conclusions of this article will be made available by the authors, without undue reservation.

## Author Contributions

XJ, SA, AB, DS, OM, and MB contributed to the conception of the approach and conducted experiments. FG and CG provided equipment and contributed to the analysis of the data. XJ, MF, and AG wrote the manuscript. MF and AG coordinated the work. All authors contributed to the article and approved the submitted version.

## Conflict of Interest

The authors declare that the research was conducted in the absence of any commercial or financial relationships that could be construed as a potential conflict of interest.

## Footnotes

1 https://www.zeiss.com/microscopy/int/downloads/zen.html

2 http://www.comstat.dk

3 http://gwyddion.net/

4 https://www.gimp.org/downloads/
