# Supplementary material for: Deleterious Effects of an Air Pollutant (NO2) on a Selection of Commensal Skin Bacterial Strains, Potential Contributor to Dysbiosis?
Source: Front Microbiol. 2020 Dec 8;11:591839. doi: 10.3389/fmicb.2020.591839 (PMC7752777; doi:10.3389/fmicb.2020.591839)
Supplement: Supplementary file 1 [file Data_Sheet_1.PDF]

Supplementary Material

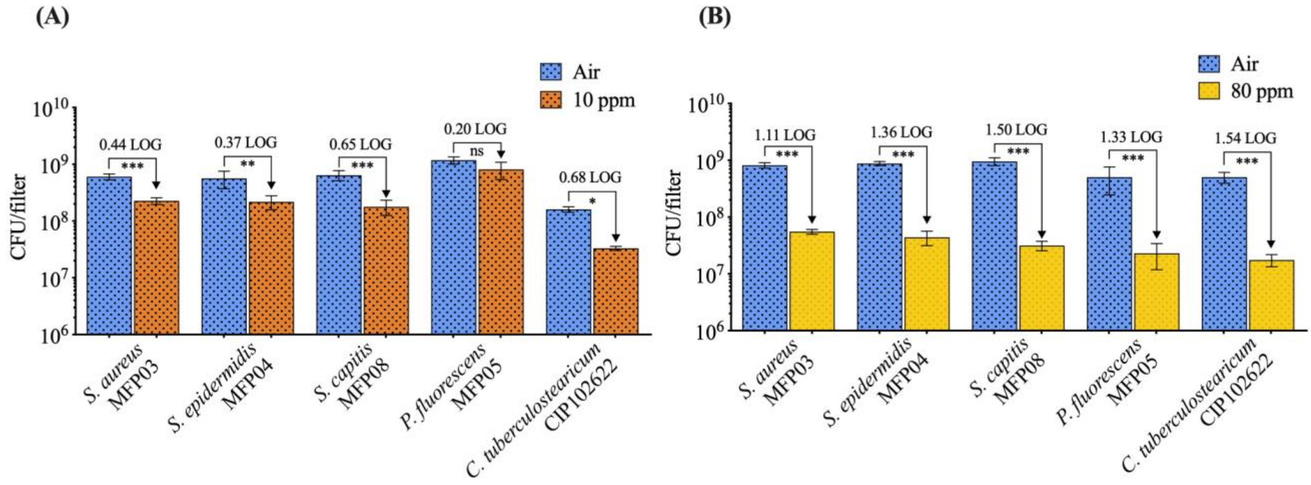

Supplementary Figure 1. Cultivability (CFU/filter) after gas exposure. For each strain and each gNO_2 concentration (10 or 80 ppm), the LOG reduction is indicated on the graph. The LOG reduction is equal to the base 10 logarithm of the ratio of the cultivability after exposure to air against that after exposure to gNO_2. Error bars show standard error of the mean of independent treatments ($n \geq 3$). Statistical significance was calculated by a ratio paired t test. (*) for $P < 0.05$, (**) for $P < 0.01$, and (***) for $P < 0.001$.

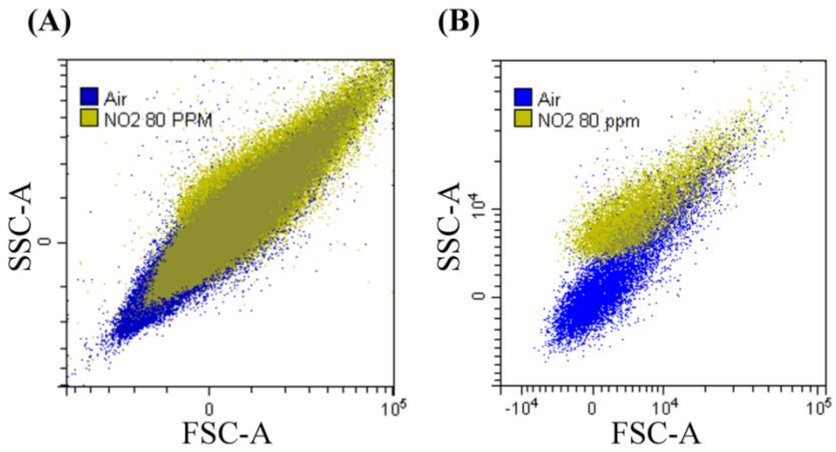

Supplementary Figure 2. Impact of 80 ppm gNO_2 on bacterial structure observed by Flow Cytometer. Dot plot overlays of FSC and SSC parameters obtained by flow cytometry analysis for air (blue) exposure and 80 ppm gNO_2 (yellow) exposure for **(A)** *S. epidermidis* MFP04 and **(B)** *S. capitis* MFP08.

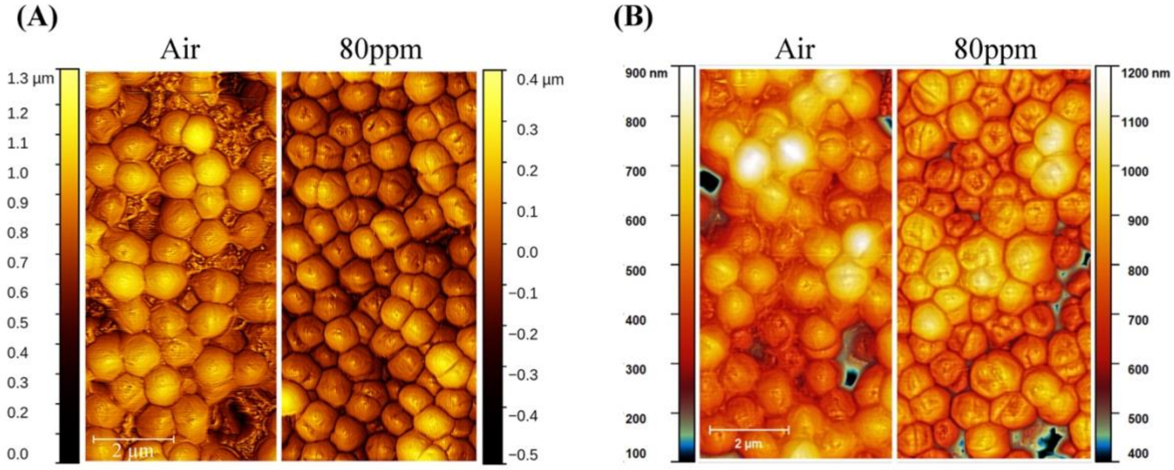

Supplementary Figure 3. Impact of 80 ppm gNO_2 on bacterial structure observed by Atomic Force Microscopy. Topographic images with overlaid local contrast images of **(A)** *S. epidermidis* MFP04 and **(B)** *S. capitis* MFP08.

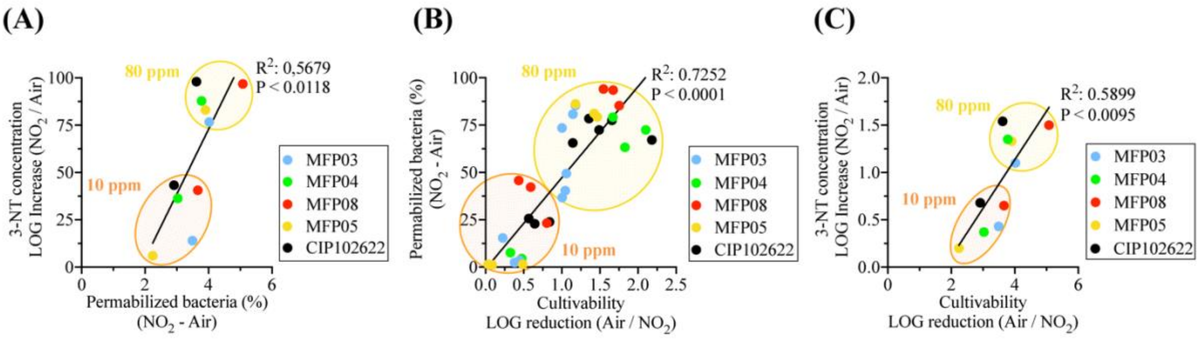

Supplementary Figure 4. Graphical representation of correlation tests. (A) Mean scatter plot of Pearson's correlation between the LOG increase of 3-NT level and percentage of permeabilized bacteria. **(B)** Scatter plot of Pearson’s correlation between permeability and cultivability. **(C)** Mean scatter plot of Pearson’s correlation between the LOG increase of 3-NT level and cultivability loss.
